# Enhanced osteopontin splicing regulated by RUNX2 is HDAC-dependent and induces invasive phenotypes in NSCLC cells

**DOI:** 10.1186/s12935-019-1033-5

**Published:** 2019-11-21

**Authors:** Jing Huang, Siyuan Chang, Yabin Lu, Jing Wang, Yang Si, Lijian Zhang, Shan Cheng, Wen G. Jiang

**Affiliations:** 1 Department of Medical Genetics and Developmental Biology, School of Basic Medical Sciences, Capital Medical University, Beijing, 100069 China; 2 Beijing Key Laboratory of Cancer & Metastasis Research, Capital Medical University, Beijing, 100069 China; 3 Department of Thoracic Surgery, Key Laboratory for Carcinogenesis and Translational Research Ministry of Education, Peking University Hospital, Beijing, 100142 China; 4 Cardiff China Medical Research Collaborative, Cardiff University School of Medicine, Heath Park, Cardiff, CF14 4XN UK

**Keywords:** OPN, Splicing, RUNX2, HDAC, EMT

## Abstract

**Background:**

Increased cell mobility is a signature when tumor cells undergo epithelial-to-mesenchymal transition. TGF-β is a key stimulating factor to promote the transcription of a variety of downstream genes to accelerate cancer progression and metastasis, including osteopontin (OPN) which exists in several functional forms as different splicing variants. In non-small cell lung cancer cells, although increased total OPN expression was observed under various EMT conditions, the exact constitution and the underlining mechanism towards the generation of such OPN splicing isoforms was poorly understood.

**Methods:**

We investigated the possible mechanisms of osteopontin splicing variant and its role in EMT and cancer metastasis using NSCLC cell line and cell and molecular biology techniques.

**Results:**

In this study, we determined that OPNc, an exon 4 excluded shorter form of *Opn* gene products, appeared to be more potent to promote cell invasion. The expression of OPNc was selectively increased to higher abundance during EMT following TGF-β induction. The switching from OPNa to OPNc could be enhanced by RUNX2 (a transcription factor that recognizes the *Opn* gene promoter) overexpression, but appeared to be strictly in a HDAC dependent manner in A549 cells. The results suggested the increase of minor splicing variant of OPNc required both (1) the enhanced transcription from its coding gene driven by specific transcription factors; and (2) the simultaneous modulation or fluctuation of the coupled splicing process that depends to selective classed of epigenetic regulators, predominately HDAC family members.

**Conclusion:**

Our study not only emphasized the importance of splicing variant for its role in EMT and cancer metastasis, but also helped to understand the possible mechanisms of the epigenetic controls for defining the levels and kinetic of gene splicing isoforms and their generations.

## Background

Lung cancer emerges as the most common cancer malignancy worldwide and is top ranked in both incidence and mortality [1]. Although current advances in chemotherapy have improved the survival of patients with non-small cell lung cancer (NSCLC), most patients die because of cancer invasion and metastasis with limited life span [2]. Growing evidence have suggested that the process of epithelial–mesenchymal transformation (EMT) is an important mechanism to trigger the invasion and metastasis of lung cancer cells and responsible for the poor prognosis of cancer patients. The current exploration on these EMT makers or EMT-TFs for their potential application in clinical diagnosis or prognosis could be of special interest for improving the treatments of lung cancers. Unfortunately, many of such attempts seemed to be cumbersome, as the known candidate molecules (*E*-*cadherin, vimentin*, *fibronectin,* etc.) or associated TFs (*ZEB1*, *SNAIL* and *TWIST*, etc.) are neither sensitive enough nor easily detected from conventional clinical samples. On the other hand, the searching for connection of currently known cancers markers for its connection to EMT regulation became an alternative solution for validating possible clinical marker indicating changes in EMT phenotype. To this end, a canonical cancer marker protein osteopontin (OPN) deserves immediate attention.

OPN is implicated in the progression of fibrosis, cancer, and other metastatic disease in multiple organ systems [3]. From recent reports, OPN was also demonstrated as a key regulator to EMT programs in cancers [4]. It was shown that OPN was able to drive EMT through specific cellular signaling pathways, and also restructure the microenvironment during EMT stages. As a secretory protein which can easily detected in the plasma, OPN is an attractive marker for its clinical values in cancer diagnosis. Extracelluar OPN proteins bound to integrin (α_v_β_3_) at the cell surface, and subsequently activated the PI_3_K/pAkt/NF-κB pathway to regulate NF-κB/ZEB-dependent EMT signals [5]. OPN was also found to upregulate hypoxia-inducible factor-1α (HIF-1α) to amplify EMT downstream signals upon TWIST activation. Ectopic expression of OPN induced upregulation of key EMT-related transcription factors, including TWIST, SNAIL, and SLUG, causing enhanced cell migration with repressed E-cadherin expression [6]. In breast cancer cells, serine phosphorylation of TWIST resulted from OPN overexpression allowed its binding to the Blymphoma Mo-MLV promoter at the insertion region 1 homolog (Bmi-1), which ultimately accelerated EMT progression in cancer cells [7].

The primary OPN transcript produces at least three major OPN splicing isoforms (OPN-SI), named OPNa, OPNb and OPNc respectively. OPNa is regarded as the full length isoform; OPNb and OPNc are the mutual excluded splicing isoforms, where the former lacks of exon 5 and the latter is missing exon 4 [8]. Several other minor isoforms of OPN with lower tissue abundance have also been identified, such as isoform 4 (OPN4) and isoform 5 (OPN5) which documented as annotated transcripts in the databases of NCBI-Unigene/Nucleotide, EBI-ENA and UniProt [9]. OPN proteins exist in different forms from either full-length or processed transcript variants, and subsequently can be further processed into various proteolytic fragments with or without chemical modifications, such as glycosylation. However, the regulation and functions of OPN-SIs remain to be obscure, especially about whether the emergence or changes in expression of certain OPN-SI is sufficient to indicate the occurrence or progress of EMT needs to be clarified.

The regulation of alternative splicing was early revealed to involve elements of specific RNA sequence and their associated factors. Recent studies started to show that epigenetic regulation not only determines the genome DNA sequence to be transcribed, but can also define how the transcript to be spliced. In eukaryotic organisms, instead of being a next step following gene transcription, splicing events occur co-transcriptionally under most circumstances. Therefore, the previous recruitment model interpreting the splicing mechanism was evolved into a kinetic model for better explaining the coupling of transcription and splicing processes. In the kinetic model, the rate of transcription could determine which cis-elements (SREs or splice sites, derived from the recruitment model) was openly accessible for spliceosomal and regulatory proteins at any defined moment. As a result, slow transcription favors the inclusion of cassette exons, whereas fast transcription in rate more likely to cause exon skipping. At the epigenetic layers, methylation of cytosines in DNA molecules, nucleosome occupancy, histone variants with modifications were implicated for altering the rate of transcription. For examples, histone acetylation on the exon–intron junctions often promotes skipping of exons, while deacetylation exerted opposite effects. Epigenetic remodeling in chromatin configuration is also thought to change the rate of transcription elongation rate, introducing fluctuation at certain regions where RBPs are recruited to affect the splicing of nascent transcripts. In fact, the epigenetic regulation on mRNA splicing indeed involve common transcription factors that are able to bind the gene promoters. These TFs not only play critical roles in priming the transcription, but also potentially interact with HDAC family members to interfere the transcription rate at the elongation stages. It was hypothesized that the activity of TFs is highly dependent to chromatin configuration in context of epigenetic modifications, which could also be the structure bases for assembling of splicing regulators.

RUNX2 is discovered as a master regulator of osteogenesis, but is abnormally expressed in several cancer types, including NSCLC [10]. Altered RUNX2 function is involved in unchecked pathways promoting tumor progression, EMT, and metastasis [11]. Among transcription factors that were able to regulate OPN expression, RUNX2 was earlier reported to modulate the expression and secretion levels of OPN proteins in osteosarcoma cells. RUNX2 was also known as a cancer-related transcription factor to promote the adhesion of endothelial pulmonary cells and the cancer lung metastasis [12]. However, whether RUNX2 could serve as a major factor contributing to the production of different OPN-SIs was not yet investigated. Certain OPN-SIs are increasingly recognized as possible markers in several types of cancers. In lung cancer patients, the major spliced OPN forms can be detected in both pleura and tumor tissues. Besides markedly upregulated OPNa, OPNc shows significant correlation with invasive behavior [13] and was found to be negatively correlated with the levels of E-cadherin and β-catenin [14]. The OPNc blood levels increased significantly as higher grade tumor was under development [15]. Recent studies strongly suggested that OPN-SI of specific types might specifically indicate certain cancer pathological features and biological functions of clinical meanings. In-depth investigation on OPN-SIs will expand the knowledge to cancer cell biology and could potentially lead to the discovery of referable cancer biomarkers.

In this study, we explored on the emergence of OPN-SIs from the epigenetic regulation of OPN total mRNA transcription, as well as the physiopathological role of OPNc on EMT development. We characterized the role of RUNX2 on the regulation of different OPN-SI expression levels. The results showed that RUNX2 was able to alter OPN splicing in dependent to HDAC activity with the involvement of splicing regulator SRSF1. We also demonstrated that OPNc posed a potent function to induce and maintain an invasive phenotype of lung cancer cells, where EMT signals was being activated. These findings are important for understanding the progression of TGF-β induced EMT in lung cancers, during which both RUNX2 and OPN, in particular OPNc as a functional splicing isoform, were upregulated. It was also suggested that inhibitors of HDACs could be potential reagents to suppress the production of total OPN and OPN-SIs, thus to alleviate the TGF-β induced EMT malignancy in lung cancers.

## Materials and methods

### Tissue culture and cell treatments

The human non-small cell lung cancer cell line A549 was obtained from the American Type Culture Collection (ATCC) (Manassas, VA). Cells were maintained at 37 °C in a 5% CO_2_ incubator in Dulbecco’s modified Eagle’s medium (DMEM) with 10% fetal bovine serum (FBS) (Biological Industries, Kibbutz Beit Haemek, Israel) and 1% penicillin–streptomycin (Keygen Biotech, Nanjing, China). Recombinant human TGF-β (R&D Systems, Minneapolis, MN) was used for treating cells at 5 ng/ml for 48 h. The HDAC inhibitor trichostatin A (TSA) (MCE, Monmouth) at 60 nM or sodium butyrate (NaB) (Sangon Biotech, Shanghai, China) at 1 mM were used for 24 h in assays for evaluating epigenetic regulation of gene expression.

### Human lung cancer specimen

A total of 92 NSCLC tissue samples and 90 matched adjacent paracancerous tissues were collected from NSCLC patients who received curative resection in Peking University Hospital. The tissue samples were treated following the SOP procedures immediately after surgical operations and stored in the Tissue Bank of Cancer Institute of Capital Medical University. Clinic-pathological information, including age, sex, histological types of tumors, TNM stage, and lymph node metastasis were recorded and registered into the patients’ database. Ethical approval for the present study was obtained from the Peking University Cancer Hospital Ethics Committee.

### Plasmid or siRNA transfection

The expression cassettes of OPN-SIs (OPNa, OPNb and OPNc) and RUNX2 were cloned individually into pENTER vectors by Vigene Bioscience Co., Ltd. (Shandong, China) and amplified in bacterial host cells. The siRNAs targeting against RUNX2 or SRSF1, shown in Table 1, were obtained from GenePharma (Shanghai, China). The siRNAs for HDAC1, HDAC2 or HDAC3 were purchased from Santa Cruz Biotechnology, Inc. (CA). The cells were either transfected with the expression plasmids or paired siRNA oligos for targeted gene using Lipofectamine™ RNAiMAX transfection Kit (Invitrogen, Massachusetts) using the vendor’s recommended protocols. Western blotting was performed to detect the resulted expression of the corresponded genes. The construct of pGL3-6xRUNX2-Luc reporter were used in A549 cells for determining the transactivation for gene transcription levels controlled by specific promoters.

**Table 1:** Primers for PCR-based assays and siRNAs used for transfection

Target	Oligonucleotide sequence
qPCR	
OPNt	F: GCCGAGGTGATAGTGTGGTT
	R: AACGGGGATGGCCTTGTATG
OPNa	F: GCCGAGGTGATAGTGTGGTT
	R: AACGGGGATGGCCTTGTATG
OPNb	F: ATCTCCTAGCCCCACAGAC
	R: AAAATCAGTGACCAGTTCATCAG
OPNc	F: TGAGGAAAAGCAGAATGCTG
	R: GTCAATGGAGTCCTGGCTGT
β-actin	F: CATTAAGGAGAAGCTGTGCT
	R: ACTGAACCTGACCGTACAGCTCGTAGCTCTTCTCCAG
GAPDH	F: GGAGCGAGATCCCTCCAAAAT
	R: GGCTGTTGTCATACTTCTCATGG
PCR	
OPN	F: AGCAGAATCTCCTAGCCCCA
	R: ACGGCTGTCCCAATCAGAAG
E-cadherin	F: TGCCCAGAAAATGAAAAAGG
	R: GTGTATGTGGCAATGCGTTC
N-cadherin	F: GACAATGCCCCTCAAGTGTT
	R: CCATTAAGCCGAGTGATGGT
Vimentin	F: CTGCAGGACTCGGTGGACTT
	R: GAAGCGGTCATTCAGCTCCT
Snail	F: TACAGCGAGCTGCAGGACTCTAAT
	R: AGGACAGAGTCCCAGATGAGCATT
Slug	F: TGATGAAGAGGAAAGACTACAG
	R: GCTCACATATTCCTTGTCACAG
Twist	F: GCCGACGACAGCCTGAGCAA
	R: CGCCACAGCCCGCAGACTTC
RUNX2	F: ACGGCAGCGGACAGCAGA
	R: TGCGGATAGCAACACAGTTCT
GAPDH	F: GGCTGCTTTTAACTCTGGTA
	R: GACTGTGGTCATGAGTCCTT
siRNA	
scramble	UUCUCCGAACGUGUCACGUTT
SRSF1	CCAACAAGATAGAGTATAATT
	TGAAGCAGGTGATGTATGTTT
RUNX2	CUCUGCACCAAGUCCUUUUTT
	GGUUCAACGAUCUGAGAUUTT

A set of minigene reporters for the splicing of OPNb and OPNc was constructed by Genechem Co., Ltd. (Shanghai, China). The synthetic sequences that match the targeted exon and the flanking introns were cloned separately into a pGL3-basic-Luc-Reporter vector immediately downstream of the ATG start codon of the luciferase gene. The splicing minigene of pGL3-OPNSIb-luc is the fusion of exon 4, initial 300 bp of intron 4, last 300 bp of intron 4, exon 5, intron 5 and exon 6 of the *Opn* gene. The pGL3-OPNSIc-luc minigene contains fused segments of the O*pn* gene in the order of exon 3, initial 300 bp of intron 3, last 300 bp of intron 3, exon 4, intron 4 and exon 5. The stop codons were engineered at the end of the alternative spliced exons, i.e. exon 4 or 5 for each of the two constructs as shown in Additional file 1: Figure S1. When cells were transfected and subjected to various treatments, luciferase reporter assay were carried out and cross-referenced with the real time PCR and western blot results.

### RNA preparation and qRT-PCR

For quantitative analyses of various OPN transcripts in NSCLC and paracancerous tissue, RNA extraction using Trizol (Life Technologies, Carlsbad), reverse transcription (RT) and qPCR were performed as previously described [16]. OPNc qPCR primer is listed in Table 1. Z-sequence on qPCR primers is 5′-ACTGAACCTGACCGTACA-3′, which is complementary to the universal Z probe used for the QPCR (TCS Biological Ltd., Oxford, UK). Real-time QPCR conditions were 95 °C for 15 min, followed by 60 cycles at 95 °C for 20 s, 55 °C for 30 s and 72 °C for 20 s. The quality of cDNA samples was verified using β-actin as a housekeeping gene.

In the quantitative analysis of OPNa, OPNb, OPNc and OPNt transcripts in A549 cells, total RNA was isolated using Trizol (Life Technologies, Carlsbad). HiScript II Q RT SuperMix for qPCR Kit (Vazyme, Nanjing, China) was used for reverse transcription. The obtained cDNA was subjected to qRT-PCR assays using NovoStart^®^ SYBR qPCR SuperMix Plus (Novoprotein, Shanghai, China). The primers for detecting OPNt, OPNa, OPNb, OPNc, SRSF1 and RUNX2 are listed in Table 1. GAPDH was used for normalization of gene expression.

### Western blotting

Western blot analysis was performed as earlier described [17]. Briefly, cell lysates were prepared and proteins were separated by 10% SDS–polyacrylamide gel electrophoresis (SDS-PAGE), and then transferred onto polyvinylidene fluoride (PVDF) filters. The probing antibodies used were as against the following antigens: E-cadherin (ab1416, mouse monoclonal antibody, 1:1000, Abcam, Cambridge, UK), N-cadherin (ab19348, mouse monoclonal antibody, 1:1000, Abcam), Vimentin.

SNAIL (ab53519, goat polyclonal antibody, 1:1000, Abcam), SLUG (sc166476, mouse monoclonal antibody, 1:1000, Santa Cruz), Twist.

OPN (ab8448, rabbit polyclonal antibody, 1:1000, Abcam), RUNX2 (ab115899, mouse monoclonal antibody, 1:4000, Abcam), SRSF1 (32–4500, mouse monoclonal antibody, 1:250, Thermo Fisher Scientific, Waltham, MA), HDAC1 (CY5154, rabbit monoclonal antibody, 1:1000, Abways, Shanghai, China), HDAC2 (CY5062, rabbit monoclonal antibody, 1:1000, Abways), HDAC3 (CY5595, rabbit monoclonal antibody, 1:1000, Abways), DDDK (M185-3L, mouse monoclonal antibody, 1:1000, MBL, Beijing, China) and GAPDH (TA-08, mouse monoclonal antibody, 1:3000, ZSGB-BIO, Beijing, China).

### Luciferase reporter assay

A total of 1 × 10^5^ cells co-transfected with splicing reporter minigene pGL3-OPNSIb-luc or pGL3-OPNSIc-luc and a pRenilla control were seeded in 24-well plates. The overexpression plasmid or siRNAs to targeting gene were also used for transfection by Lipofectamine™ 2000 reagent (Invitrogen). After continuous culture for 48 h, the firefly and renilla luciferase activities of the collected lysates were measured with a Dual-Luciferase Reporter System (Promega, Madison, WI, USA).

### Wound-healing assay

Cells were seeded in 6-well plates until formed a confluent monolayer. A straight scratch line was made in the monolayer using pipette tips in each well. The closing of the gaps was observed under an inverted microscope during the incubation period. Digital images were acquired at each time point. The distance between the boundary edges of cell growth was measured as the quantifiable index for cell migration using ImageJ software.

### Cell invasion assay

Transwell chambers adapted with a 6.5 mm diameter polycarbonate filter insert (pore size 8 μm) (Corning, Corning, NY, USA) were pre-coated with 50 μg/insert of Matrigel (Corning). Cells were planted at a density of 2 × 10^4^ cells/chamber. After incubation for 48 h, the cells invaded through the Matrigel were fixed, stained. Quantification and statistics was performed as described before [18].

### Statistical analysis

The computer program Prism 6 (GraphPad Software, Inc., La Jolla, CA, USA) was used for statistical analyses. The association between gene expression and clinical factors was analyzed using Mann–Whitney U test. The Student’s *t* test was used to compare cell functions between paired groups. The analysis of variance (ANOVA) was used to determine the statistical significance of data in multiple groups. The overall survival rates were calculated using the Kaplan–Meier method. Cases of *p*-value < 0.05 was defined as statistically significant.

## Results

### Correlated upregulation of OPN and RUNX2 during TGF-β induced EMT in A549 NSCLC cells

**Fig. 1:**
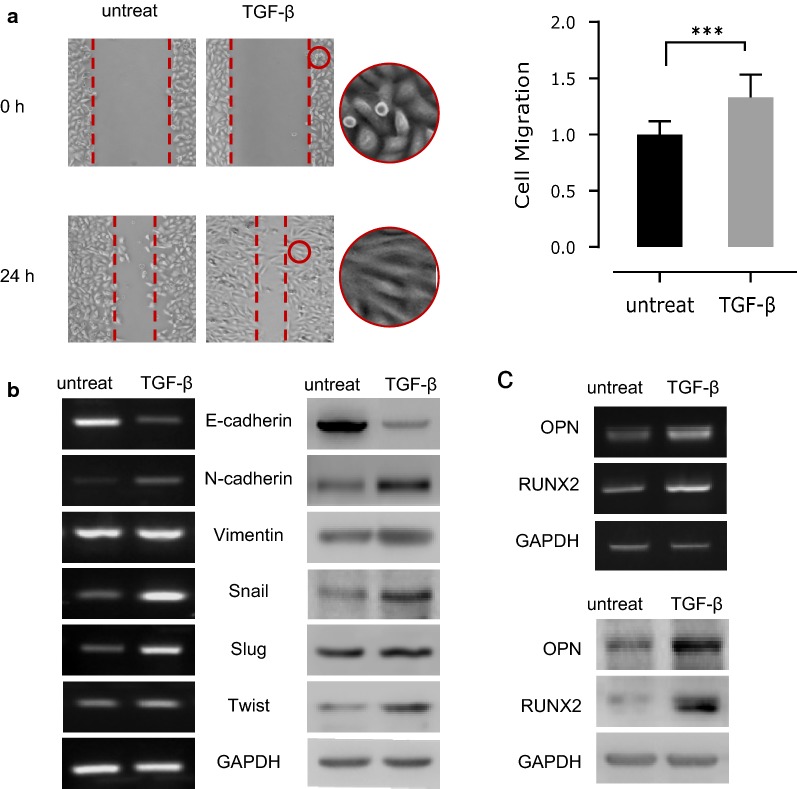
Upregulation of RUNX2 associated with increased OPN expression during the EMT progression in TGF-β treated A549 cells. **a** TGF-β treatments for 48 h increased cell migration. **b** TGF-β treatments induced significant changes in the expression of major EMT marker genes at both mRNA (left) and protein (right) levels. **c** The mRNA (upper) and protein (lower) expression of OPN and RUNX2 were synchronically increased following TGF-β treatments

We used TGF-β to treat NSCLC cells for inducing a migratory phenotype in A549 cells, as it is known that the development of EMT could be promoted by TGF-β in lung tumor cells [19]. Exposed in TGF-β (5 ng/ml) for 48 h, the cells exhibited increased mobility comparing to the untreat controls (*p *< 0.01) (Fig. 1a). Besides, a significant percentage in TGF-β treated cells reshaped into a mesenchymal-like morphology from their native cobblestone epithelial type as in the controls. Such morphological changes towards fibroblast-like states are often an indication for EMT progression, therefore, we tested the expression of several EMT markers for their expression following TGF-β treatments in A549 cells through RT-PCR and western blotting. The results showed that both N-cadherin and Vimentin, as EMT signature molecules, were upregulated, accompanied with markedly downregulated E-cadherin. Other EMT-specific transcription factors, such as SNAIL, SLUG and TWIST were also upregulated, comparing to the untreat A549 cells (Fig. 1b). As expected, the significantly increased OPN was observed during EMT progress. Meanwhile, the expression of RUNX2, a TF could recognize the conserved motif at a distal region of the *Opn* gene promoter, was found to be increased at both mRNA and protein levels in TGF-β induced A549 cells (Fig. 1c). These results suggested that during TGF-β induced EMT in NSCLC cells, the associated upregulation of RUNX2 and OPN might be functionally important for the development, as well as the progress of EMT.

### RUNX2 enhanced TGF-β induced *Opn* gene expression and promoted OPN alternative splicing

**Fig. 2:**
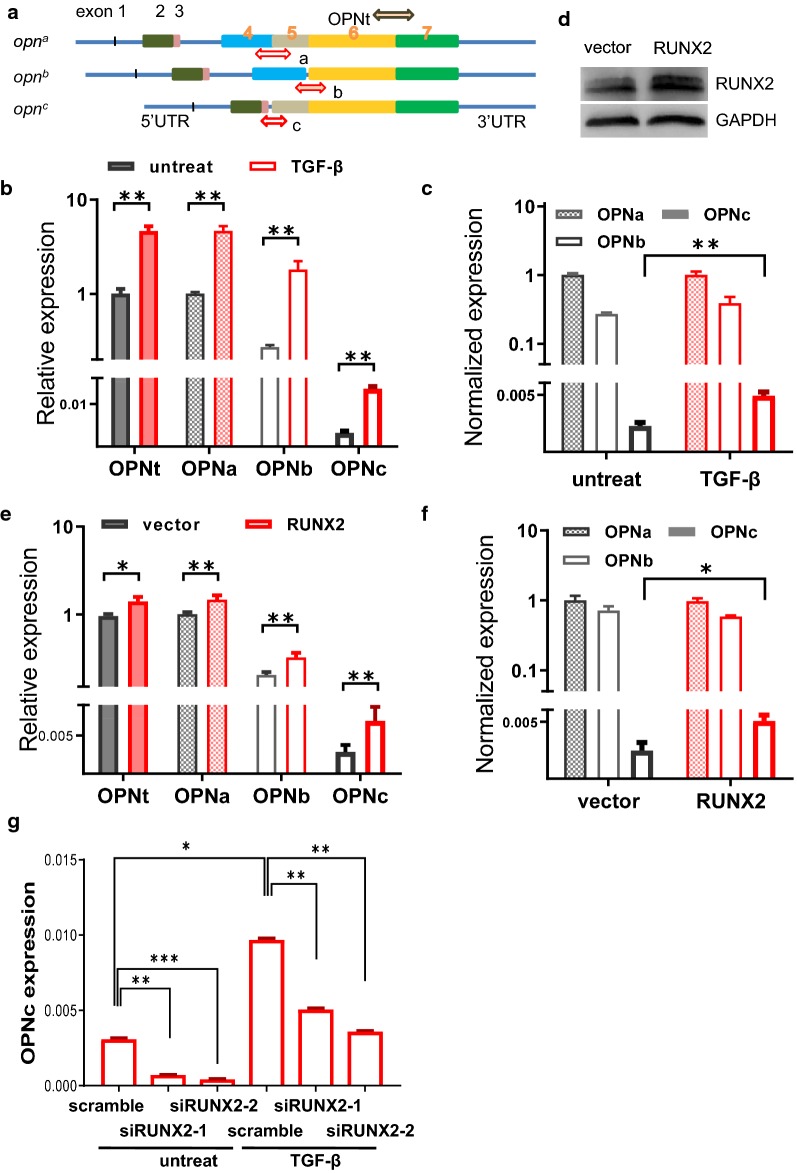
RUNX2 overexpression enhanced the expression of OPN, especially of splicing isoform c. **a** Schematic illustration of isoform-specific primers used for the quantitative analyses of OPN-SIs. **b** TGF-β induction increased the *opn* transcript levels unproportionally among different splicing isoforms. **c** TGF-β preferentially promoted OPNc splicing in A549 cells. **d** Western blot of RUNX2 in A549 cells transfected with an overexpression plasmid. **e** Overexpression of RUNX2 altered the OPN-SIs and OPNt mRNA levels. **f** RUNX2 overexpression selectively increased OPNc transcript levels. **g** Knockdown of RUNX2 expression attenuated TGF-β induced *opn* expression in A549 cells

To address whether the expression of OPN splicing variants following TGF-β induction is affected with the changes in RUNX2 level and functions, we detected the mRNA levels, as well as the abundance of major OPN-SI species using qRT-PCR with specific primers probing for splicing junctions (Fig. 2a). The overexpression of wild type or dominant negative RUNX2 was also used for transfection to verify the OPN-SI expression under the influence of RUNX2 transcription regulatory activities. The results showed that the mRNA levels of OPNt and OPNa, OPNb, OPNc were increased in the TGF-β induced A549 cells (Fig. 2b). Noticeably, the normalized abundance of OPNc, calculated using the ratio of OPNc to OPNt transcripts, was significantly increased by 2.5-fold over the control cells (Fig. 2c). The expression level of RUNX2 was considerably increased in the RUNX2-overexpressing A549 cells (Fig. 2d). In comparison with the vector control group, overexpressing RUNX2 resulted in a significant change in the expression levels of OPN-SIs (Fig. 2e). The normalized abundance of OPNc was increased by approximately 2.3-fold, whereas no expression change was found in OPNa and OPNb (Fig. 2f). When cells transfected with a RUNX2^R131G^ mutant, which lacks the binding ability to OPN promoter for transactivation activity (as shown from the reporter assays), responses in the mRNA level changes in OPNt and OPN-SIs were abolished (Additional file1: Figure S2). Consistently, the TGF-β induced *opn* expression was also found significantly compromised in the RUNX2 knockdown A549 cells (Fig. 2g). These data suggested that TGF-β not only induced the mRNA transcription of *Opn* gene, but also regulated the outcome of alternative splicing of *opn* mRNA. The transcription factor RUNX2 of its increased levels in TGF-β induced EMT cells could be involved in the splicing of OPN-SIs, and the RUNX2 regulation on OPN splicing was possibly require the binding of RUNX2 to OPN promoter at the interaction motif sites. Among the major forms of OPN-SIs, a less abundant OPNc appeared to be a more sensitive indicator for representing the regulating role of TGF-β on OPN splicing in NSCLC cells.

### The splicing of OPNb and OPNc required the splicing regulatory factor SRSF1

**Fig. 3:**
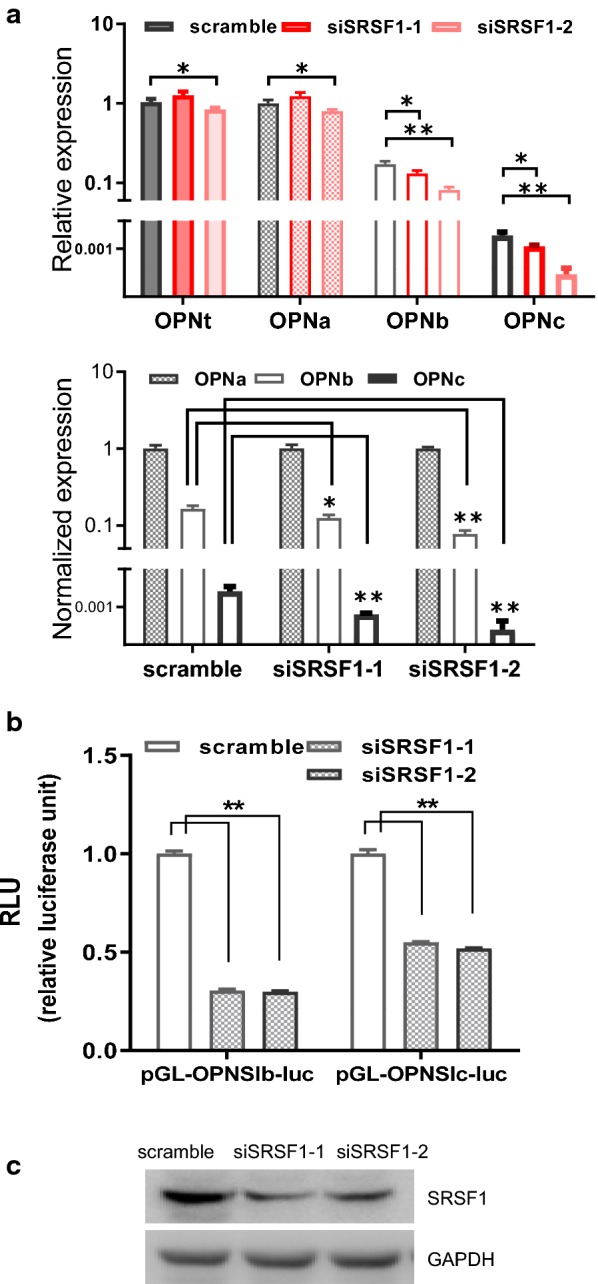
Knock down of SRSF1 reduced mRNA levels of OPN-SIs. **a** RNAi of SRSF1 decreased OPNc levels in A549 cells. **b** Inhibition of OPNb and OPNc splicing from reporter assays following SRSF1 siRNA transfection. **c** Western blot of SRSF1 in A549 cells transfected with targeted siRNAs

The splicing factor SRSF1 is a crucial family member of SR proteins. SRSF1 was known to bind exonic splicing enhancers to promote splicing [20]. It has been reported that SRSF1 was able to regulate EMT through disrupting the alternative splicing of key tumor associated genes, including Ron proto-oncogene [21], PRRC2C [22], and MKNK2 [23, 24], to modulate lung cancer progression. To investigate the role of SRSF1 on OPN alternative splicing, we tested if changes of SRSF1 expression could affect OPN splicing in A549 cells. Knocking down of SRSF1 (Fig. 3c) through the transfection with SRSF1 targeted siRNAs in A549 cells markedly reduced the splicing products of OPNb and OPNc from the isolated total RNAs (Fig. 3a) and in the splicing reporter assay (Fig. 3b). Therefore, SRSF1 is able to boost the splicing of OPNb and OPNc both in genomic transcription coupled splicing and the naked RNA from plasmid transcription.

### RUNX2 increased OPNc splicing in a HDAC dependent manner

**Fig. 4:**
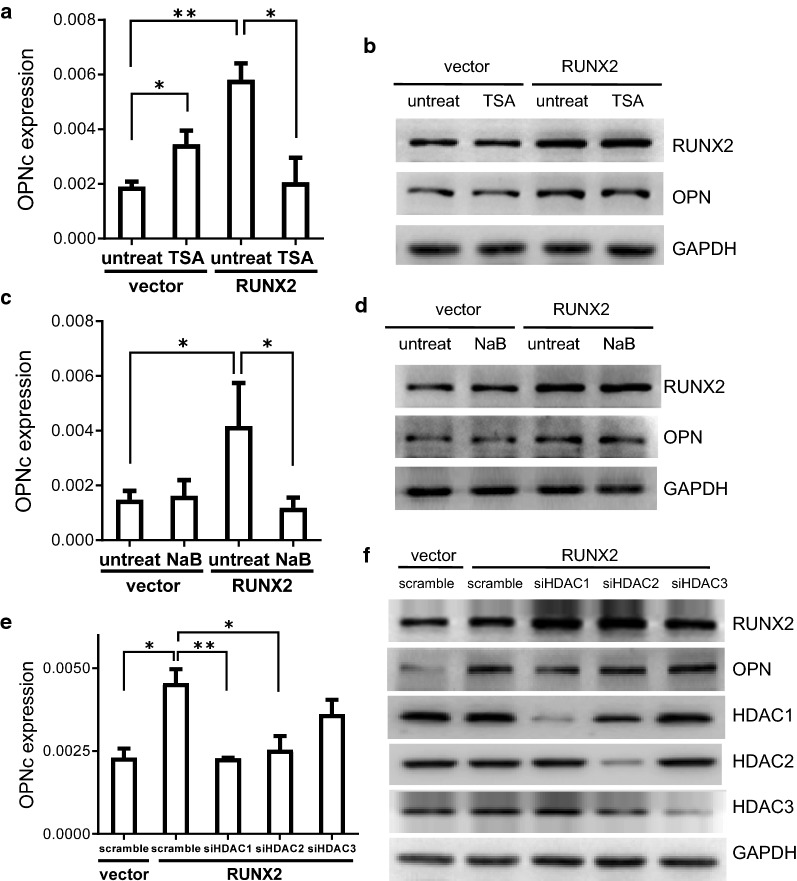
RUNX2 dependent OPNc splicing required normal activities of HDAC1 or HDAC2. **a** Treatment of A549 cells with HDACs inhibitor TSA suppressed OPNc splicing induced by RUNX2 overexpression. **b** Western blot of RUNX2 and OPN in RUNX2 overexpressed A549 cells following TSA treatment. **c** Inhibition of HDAC1 activity by NaB deprived RUNX2 induced OPNc splicing. **d** Western blot of RUNX2 and OPN in RUNX2 overexpressed A549 cells treated with NaB. **e** Knockdown of HDAC1 or HDAC2, but not HDAC3, decreased RUNX2-induced OPNc splicing. **f** Western blots of HDAC1, HDAC2 and HDAC3 from A549 cells transfected with the targeted siRNAs

Histone deacetylases (HDACs) repress gene transcription by deacetylating histone and non-histone proteins [25]. HDACs induce chromatin changes and are involved in regulation of alternative splicing [26]. RUNX2 were found to cooperate with HDACs, where RUNX2 mediated HDAC-dependent transactivation [27]. Whether the cooperative interaction of RUNX2 with HDACs contributed to the alternative splicing of OPN, especially in cancer cells following TGF-β induction, was not known to date. We treated A549 cells with widely used HDAC inhibitors TSA or NaB and found that the enhancement of RUNX2 on OPNc splicing was reduced (Fig. 4a–d). Using siRNAs to knockdown specific HDAC isoforms, we determined that HDAC1 and HDAC2 were responsible for mediating the splicing of OPNc, with the former to be more important. As compared to the scramble control group, the levels of OPNc was reduced to about 50% or 56% respectively following cotransfection of RUNX2 with siHDAC1 or siHDAC2 (Fig. 4e). Knockdown of HDAC3 did not show significant effect on the splicing of OPNc. These results suggested that with the dependence to HDAC1 or HDAC2, RUNX2 indeed participated and increased OPNc alternative splicing in A549 cells. In view of the fact that the EMT process is often accompanied with extensive epigenetic changes, we postulate that epigenetic regulation can be a fundamental mechanism allowing preferential expression of spliced transcript related to progression. Other epigenetic factors might also be considered in the emergence of OPNc splicing for the determination of selectivity.

### OPNc overexpression exhibited a potent role on the development of invasive cell phenotype and promoted EMT in A549 cells

**Fig. 5:**
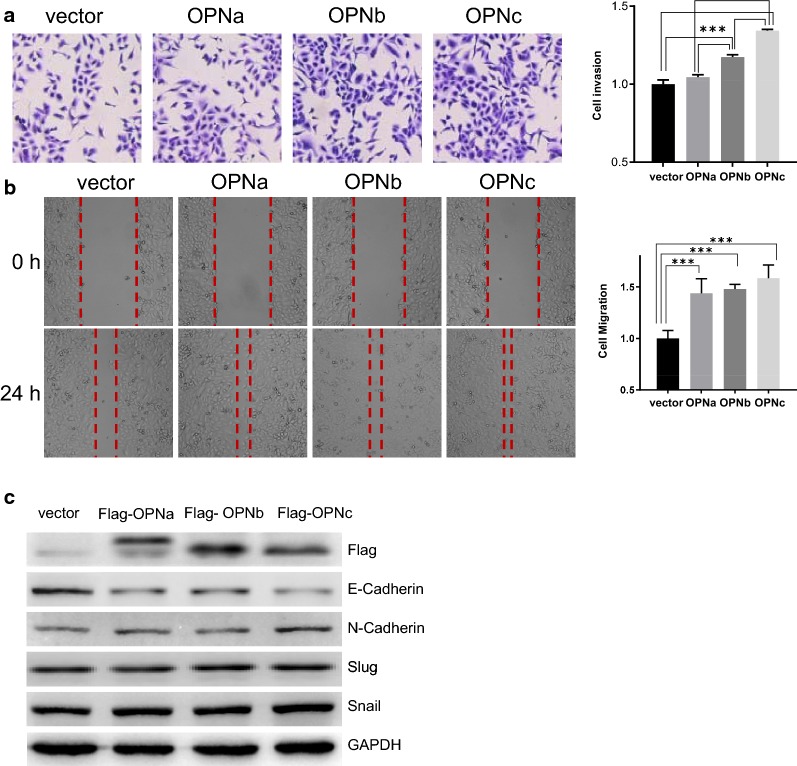
OPNc overexpression was most potent to induce an invasive phenotype in A549 cells among OPN-SIs. **a** Overexpression of OPNb and OPNc significantly increased the invasiveness of transfected cells. **b** Overexpression of OPN-SIs promoted cell mobility and migration. **c** Immunoblots of EMT marker proteins in cells with overexpression of OPN-SIs

In order to disseminate the effect of different OPN-SIs on cellular functions, we performed migration and invasion assays in A549 cells transfected with OPNa, OPNb or OPNc expression plasmids (Fig. 5c). In cell invasion assays, the results showed that OPNb or OPNc overexpression promoted the invasion of A549 cells by 120% and 140%. No significantly difference was found in OPNa overexpressed cells compared to the controls (Fig. 5a). Overexpression of either of the three OPN-SIs could lead to the increase in cell migration by 140%, 150% and 170% respectively (Fig. 5b). These results demonstrated OPNc displayed a more potent effect to induce EMT-like phenotypes in A549 cells than OPNa and OPNb, as shown in the western blots of E-cadherin and N-cadherin as EMT marker proteins. However, the differences in the expression of Snail and Slug were not appeared significant even under OPNc overexpression conditions (Fig. 5c).

### Increase of OPNc splicing and cell migration is HDAC-dependent in SK-MES-1 cells under TGF-β induction

**Fig. 6:**
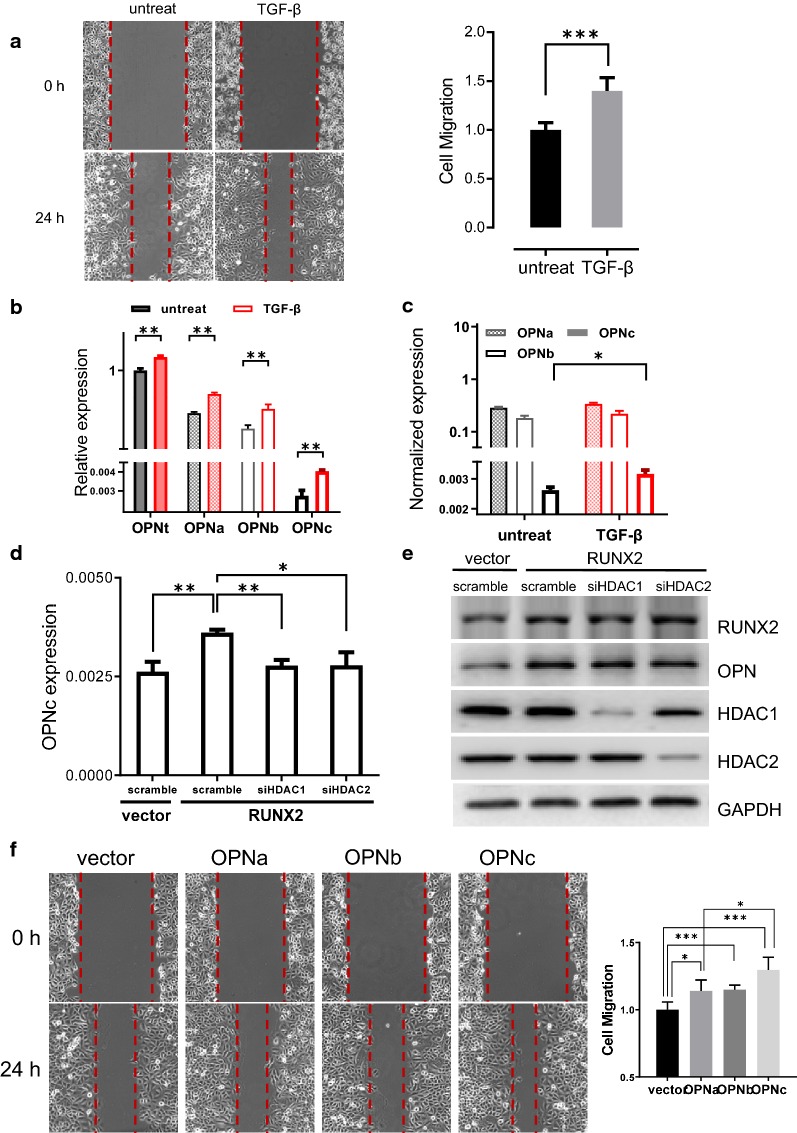
TGF-β induced OPNc expression enhanced the mobility of SK-MES-1 cells with a dependence to HDACs. **a** TGF-β treatments for 48 h increased the invasiveness of SK-MES-1 cells. **b** TGF-β induction increased OPNt and OPN-SIs levels with a preference to OPNc. **c** TGF-β promoted OPNc splicing with most significance in SK-MES-1 cells. **d** Knockdown of HDAC1 or HDAC2 significantly decreased the RUNX2-induced OPNc splicing. **e** The HDAC1 and HDAC2 expression were markedly reduced in the SK-MES-1 cells transfected with the targeted siRNAs. **f** Overexpression of OPN-SIs significantly promoted the migration of SK-MES-1 cells

To verify our findings on the role of TGF-β on OPN splicing, we selected SK-MES-1 cells, a different type of NSCLC cell of squamous cell origin, and treated with TGF-β (5 ng/ml) for 48 h. The cells exhibited increased mobility comparing to the untreat controls (*p *< 0.001) (Fig. 6a). Following TGF-β induction, the mRNA levels of OPNt and OPNa, OPNb, OPNc were increased, except the changes in transcript levels were less significant as observed in A549 cells (Fig. 6b). Among all the OPN-SIs, OPNc remained to be the most increased isoform (Fig. 6c). We then directly moved on to evaluate the role of HDACs on OPN expression and splicing by co-transfecting siHDAC1 or siHDAC2 with RUNX2 in SK-MES-1 cells. When HDAC1 was knocked down, the levels of OPNc was reduced to approximately 77%. Similar results of about 75% was observed in HDAC2 RNAi samples as compared to the scramble control group (Fig. 6d, e). In Fig. 6f comparing the effect of OPNa, OPNb and OPNc on cell migration, OPNc was again demonstrated for most significant role to promote cell mobility in SK-MES-1 cells. These results were consistent with our observation in A549 cells, and therefore indicated that the increase of TGF-β induced cell migration involved enhanced OPNc splicing, meanwhile the OPNc splicing was regulated by RUNX2 and appeared to be HDAC-dependent. Such regulatory scheme could be a common mechanism in different cancers and to affect cancer cell migration.

### Clinical association of OPN-SIs in NSCLC patients

**Fig. 7:**
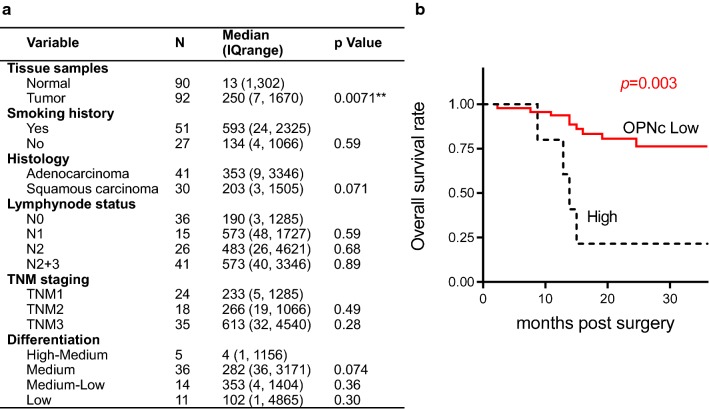
OPNc expression in association with clinical features of NSCLC patients. **a** Demographic information of collected NSCLC tissues and statistics from quantitative PCR analyses of OPNc expression. **b** Overall survival of patients with NSCLC cancer in OPNc high and OPNc low groups

Following the exact protocol to determine OPNa levels [16], we quantified the transcript abundance of OPNb and OPNc for using samples from the same tissue bank. A significantly increased level of OPNc (*p *= 0.0071) was found in the in cancer group (Fig. 7a). Noticeably, the elevated level of OPNc appeared to be more frequent in samples with low tumor differentiation, positive lymph node metastasis and high TNM stage, despite not reached for a statistical significance among these groups. When the NSCLC tissues were classified into two subgroups according to the median OPNc level, the high OPNc group was demonstrated a shorter overall survival index as compared to the low OPNc patients (Fig. 7b). This could be a piece of supporting evidence for OPNc to be used as a marker for EMT emergence and progress, which indicates more malignant features of NSCLC.

## Discussion

The role of alternative splicing (AS) during EMT has increasingly been recognized recently. AS regulates a broad spectrum of genes involved in cancer progression and metastasis. AS variants orchestrate several important process defining EMT phenotypes, including cell–cell contacts, polarity and cytoskeleton organization, and differentiation. Under many circumstances, the pivotal role of AS regulation in tumor plasticity is underscored for its complex consequence, where the rapid shifts of the expression of protein isoforms may exhibit distinct functions and are affected by many factor. However, loss of splicing fidelity indeed resulted in the production of the isoforms of various proteins controlling numerous facets of cancer. Although changes in AS occur during EMT, only a small portion of specific splicing events are known to functionally contribute to EMT and its regulation, such as the splicing of CD44, FGFR2 and Exo70 [28–30]. In the present study, a notable EMT associated splicing isoform OPNc was identified and verified for its increased presence in TGF-β induced A549 cells (Fig. 2). In NSCLC cancer tissues, it was also indicated that high level expression of OPNc significantly associated with the shorter overall survival of patients (Fig. 7), but not OPNa or OPNb (Additional file 1: Figure S3). OPNc was demonstrated to play a stronger role to promote the capability of cell migration and invasion in A549 cells, which was required during the development and advancing of EMT (Fig. 5). These data helped to explain the fact that the patients with lower tumor differentiation, more lymph node metastasis and higher TNM stages tends to exert high levels of OPNc transcripts (Fig. 7). Our results suggested that OPNc expression could be an important marker to indicate the development of EMT and the malignant progression of NSCLCs.

The splicing isoforms of OPN have been connected to be cancer progression in several tumors, yet the understanding of the mechanism for their generation and regulation are still very poor. Splicing is reported to involve the interaction between RNA binding proteins (RBPs) via presumably the cognate splicing regulatory sequence elements (SREs) in the premature mRNAs. A major class of splicing factors that control splice site recognition is the families of Serine/Arginine-rich (SR) proteins, which interact with heterogeneous nuclear ribonucleoproteins (hnRNPs). As a well characterized factor, SRSF1 was suggested to play key driver functions in solid tumors. Considering the possibility that different types of splicing events might recruit specific regulators, such as the finding in FLNB mediated EMT of its FOXC1 dependent skipping of exon 30 [31], we investigated the role of SRSF1 for the splicing of *Opn* gene in NSCLC cells (Fig. 3).

Alternative splicing by its nature is a tightly controlled process, despite the types of splicing events and the outcome in splicing variants are in great varieties, especially during EMT where the selection alternative splice site, choices for retaining introns or using alternative exons can be rather sophisticate. A subset of genes spliced with exclude exons and switched from longer to shorter isoforms in EMT cells, such asSLC37A2, KIF13A, FLNB, and MBNL1, while others showed a greater degree of exon inclusion, e.g. PLEKHA1, MLPH, ARHGEF11, CLSTN1 and PLOD2 [32]. Exon inclusion events might require remodeler A, vs. exon exclusion events, which might require remodeler B. Alternatively, there might be certain transcription factors that can specifically target different sets of chromatin remodelers to different genes, e.g. inhibition of the E-cadherin promoter transcription with ZEB1 expression increases E-cadherin aberrant splicing. RUNX2 belongs to the RUNX family, plays a role in several tumor tissues, including pancreatic cancer, breast cancer, ovarian epithelial cancer, prostate cancer, and osteosarcoma [33–37]. By analyzing public databases for recurrent RUNX2 genetic and epigenetic modifications in lung cancer, we found that the most common RUNX2 genetic alteration that exists in transcription upregulation is, followed by genomic amplification, nucleotide substitution and multiple changes (Additional file 1: Figure S4). RUNX2 was reported to modulate expression and secretion of the OPN in osteosarcoma cells to promote adhesion to endothelial pulmonary cells and lung metastasis. However, the transcription regulation of RUNX2 on OPN in lung cancer is still not clear. There is no report about the regulating role of RUNX2 on splicing yet. In the present study, it was found that RUNX2 not only enhanced the mRNA transcription of *Opn* gene, but also regulated the AS of OPN (Fig. 2).

The kinetic coupling model for splicing regulation partly explains the role of transcription factors for influencing the rate of transcription elongation which leads to the occurrence of alternative splicing in many occasions. There is also additional evidence demonstrating transcription factors interfere the splicing events through directly interacting with the spliceosome, or by recruitmenting additional regulatory factors. As the most populated chromatin remodelers, HDACs are thought to be and are demonstrated as functional epigenetic modulators to bridge the role of transcription factors in both of the transcription and splicing process. It was reported that RUNX2-induced *Opn* gene expression was under HDAC1-mediated regulation in C3H10T1/2 cells. Additionally, HDAC1 and the non-phosphorylated form of HDAC2 were found to co-precipitate with SRSF1, as shown by mass spectrometry followed by immunoprecipitation [38]. The present studies suggested that the dependence of HDAC1 and HDAC2 on OPNc splicing induced by RUNX2 overexpression (Fig. 4) possibly revealed the importance of HDACs for coordinating the couple events of transcription and splicing in eukocytes. A splicing complex maintained dynamically by SRSF1 and HDACs would be sensitive to the binding of RUNX2 to *Opn* promoter, as these sharing for the same pool of HDACs. The binding of RUNX2 to *Opn* promoter for activation of *Opn* transcription which was repressed by the interaction with HDACs, could redistribute and shunt the HDACs from the upstream to the splice sites and interact with SRSF1, leading to the increase of *Opn* splicing. The epigenetic mechanisms should be emphasized in the process of mRNA splicing especially in the transcription coupled splicing. The role of HDAC family members could be dominant in the regulation of alternative splicing. On top of upregulating the gene transcription following the transactivation of its promoter, such mechanisms are able to further translate a physiological stimulation, such as TGF-β induction, into a response to selectively amplify specific splicing variant species and producing corresponding protein isoforms for developing certain phenotypes. For EMT in lung cancer cells, the increased expression of OPNc not only directly promoted the invasiveness of cells, but also could be a solution to maintain the continuation of EMT development by attenuating or circumventing the dominance of TF controls on gene expression, which tends to be pro-proliferative and often fluctuated in cancers.

## Conclusion

In conclusion, our study discovered that the selective splicing of OPNc could be regulated by RUNX2 or SRSF1 in a HDAC depending manner. The findings improved the understanding about how OPNc appeared to be a better-performed marker to indicate the development of EMT in NSCLC. The phenotype changes in migration and invasion for cells undergo EMT involve complex interactions of transcription factors and epigenetic proteins, which lead to the spectrum changes in splicing variants, as well as the selective production of specific SIs. The epigenetic mechanisms for the regulation of gene expression will also be important for the modulation of alternative splicing, at least the role of HDAC family members should be emphasized in related studies on EMT and cancer metastasis.

## Supplementary information

**Additional file 1: Figure S1.** The design of OPN splicing minigenes. The splicing minigene cassette of pGL3-OPNSIb-luc contains the fusion of exon 4, initial 300 bp of intron 4, last 300 bp of intron 4, exon 5, intron 5 and exon 6 of the *Opn* gene at upstream of firefly luciferase CDS. Similarly, the pGL3-OPNSIc-luc minigene contains fused segments of the O*pn* gene in the order of exon 3, initial 300 bp of intron 3, last 300 bp of intron 3, exon 4, intron 4 and exon 5. The stop codons were engineered at the end of the alternative spliced exons, i.e. exon 4 or 5 in each of the two constructs. **Figure S2**. The mRNA levels of OPN and OPN-SIs were not influence by overexpression of RUNX2 mutant without the binding activity for the transactivation of OPN promoter. **a** Western blots of wild type RUNX2 or RUNX2^R131G^ mutant in transfected A549 cells. **b** Reporter assays using 6 × RUNX2 driven luciferase expression for the detection on the transcriptional activity of RUNX2 and RUNX2^R131G^ mutant. **c** The mRNA expression of OPNt, OPNa, OPNb and OPNc were not significantly changed following transfection of RUNX2^R131G^ mutant. **d** No significant difference was observed comparing the normalized mRNA abundance of OPNa, OPNb and OPNc in A549 cells tranfected with the RUNX2^R131G^ overexpression plasmids. **Figure S3**. The overall survival of patients with NSCLC tissues analyzed by OPNa and OPNb expression levels. **a** The overall survival of NSCLC patients by OPNa levels. **b** The overall survival in association with OPNb levels. **Figure S4**. Genetic alterations detected of human RUNX2 gene in lung cancers as documented in the public databases. **a** The data on gene mutation, amplification and multiple alterations of RUNX2 retrieved from cBioPortal as filtered in lung adenocarcinoma and lung squamous cell carcinoma. **b** The statistics of RUNX2 expression in lung adenocarcinoma using data collected in TCGA database.

## Data Availability

All the data generated or analysed in this study are included in the manuscript or the additional file.
