# Supplementary figures and images for: Enhanced osteopontin splicing regulated by RUNX2 is HDAC-dependent and induces invasive phenotypes in NSCLC cells
Source: Cancer Cell Int. 2019 Nov 21;19:306. doi: 10.1186/s12935-019-1033-5 (PMC6873507; doi:10.1186/s12935-019-1033-5)

Figure S1

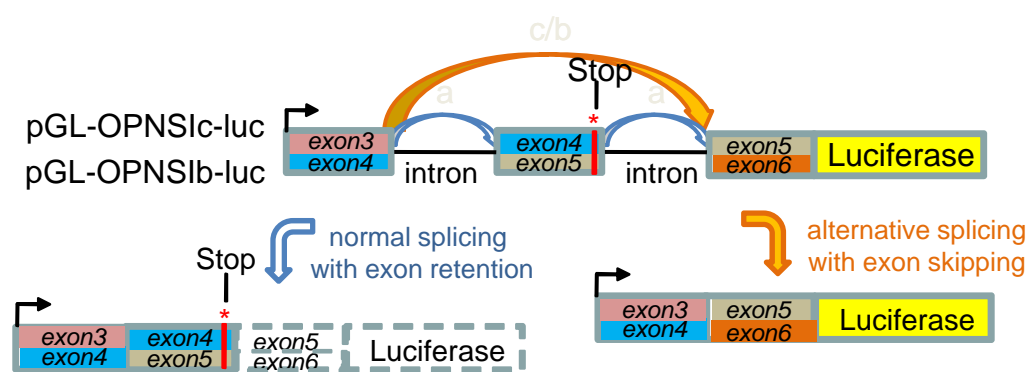

Figure S2

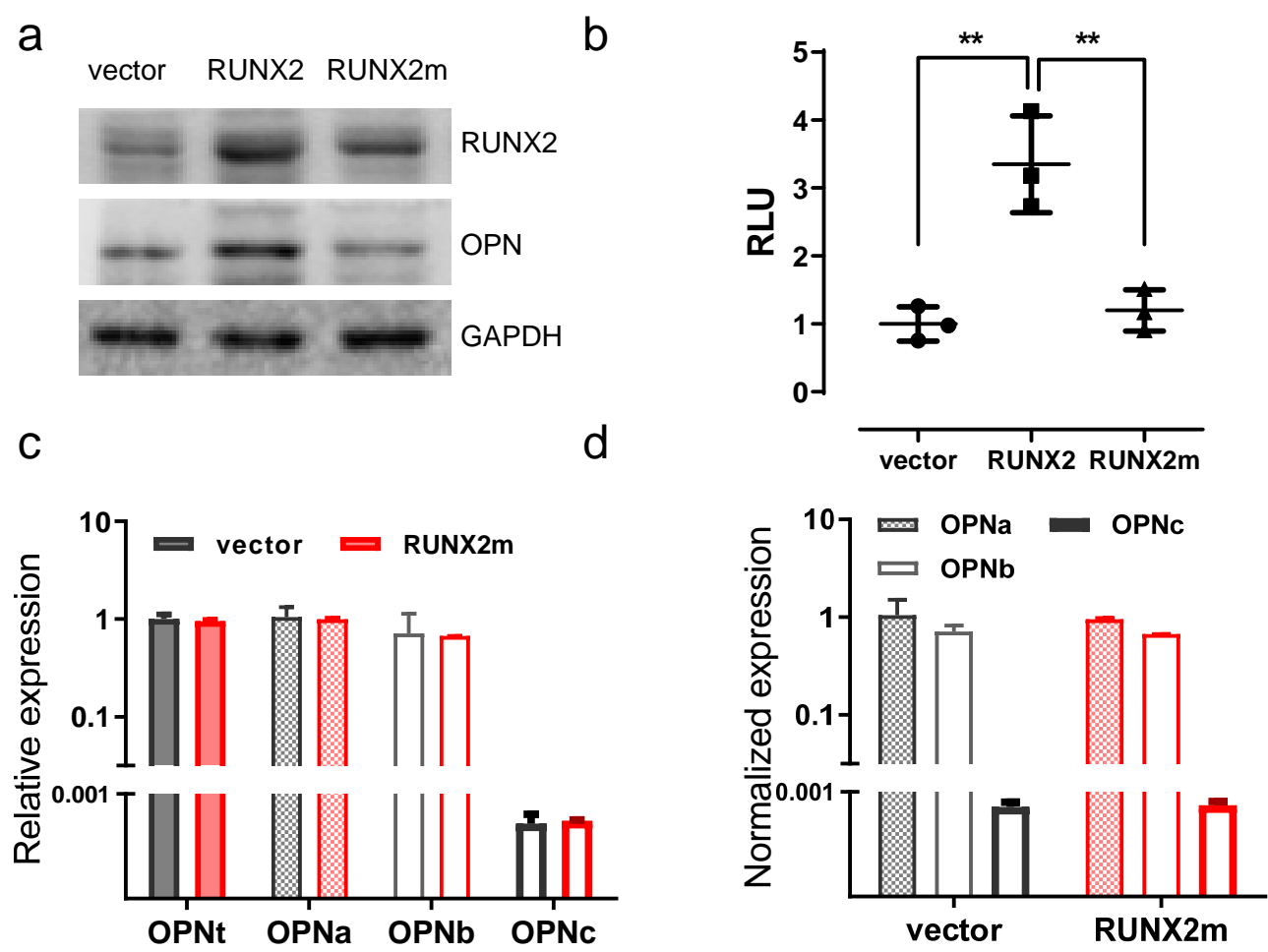

Figure S3

a

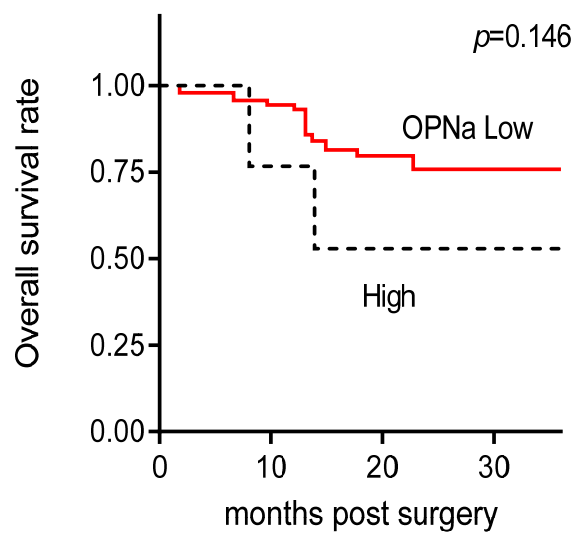

b

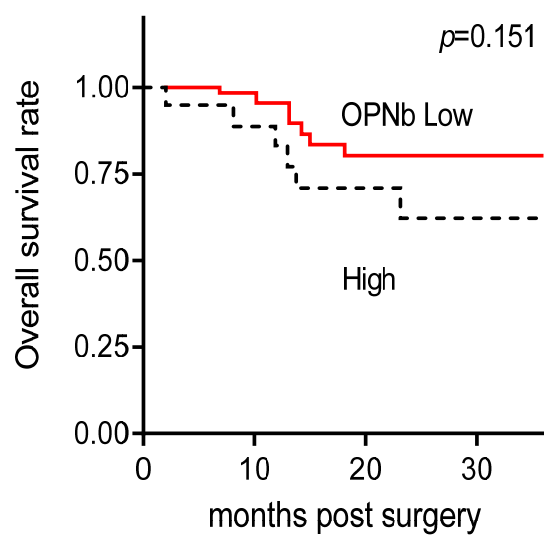

Figure S4

a

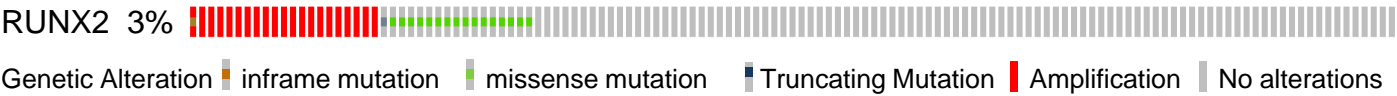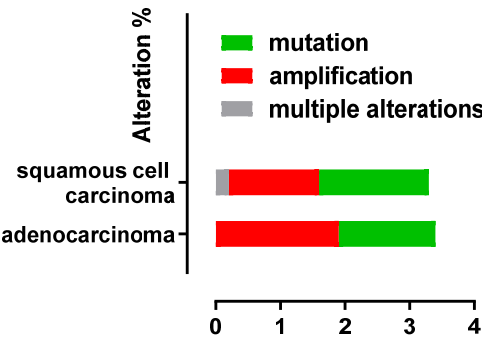

b

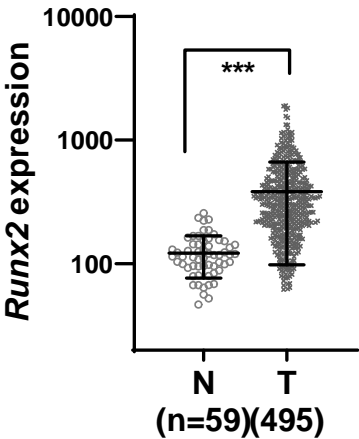

Supplement: Supplementary file 1 — Additional file 1: Figure S1. The design of OPN splicing minigenes. The splicing minigene cassette of pGL3-OPNSIb-luc contains the fusion of exon 4, initial 300 bp of intron 4, last 300 bp of intron 4, exon 5, intron 5 and exon 6 of the Opn gene at upstream of firefly luciferase CDS. Similarly, the pGL3-OPNSIc-luc minigene contains fused segments of the Opn gene in the order of exon 3, initial 300 bp of intron 3, last 300 bp of intron 3, exon 4, intron 4 and exon 5. The stop codons were engineered at the end of the alternative spliced exons, i.e. exon 4 or 5 in each of the two constructs. Figure S2. The mRNA levels of OPN and OPN-SIs were not influence by overexpression of RUNX2 mutant without the binding activity for the transactivation of OPN promoter. a Western blots of wild type RUNX2 or RUNX2R131G mutant in transfected A549 cells. b Reporter assays using 6 × RUNX2 driven luciferase expression for the detection on the transcriptional activity of RUNX2 and RUNX2R131G mutant. c The mRNA expression of OPNt, OPNa, OPNb and OPNc were not significantly changed following transfection of RUNX2R131G mutant. d No significant difference was observed comparing the normalized mRNA abundance of OPNa, OPNb and OPNc in A549 cells tranfected with the RUNX2R131G overexpression plasmids. Figure S3. The overall survival of patients with NSCLC tissues analyzed by OPNa and OPNb expression levels. a The overall survival of NSCLC patients by OPNa levels. b The overall survival in association with OPNb levels. Figure S4. Genetic alterations detected of human RUNX2 gene in lung cancers as documented in the public databases. a The data on gene mutation, amplification and multiple alterations of RUNX2 retrieved from cBioPortal as filtered in lung adenocarcinoma and lung squamous cell carcinoma. b The statistics of RUNX2 expression in lung adenocarcinoma using data collected in TCGA database. [file 12935_2019_1033_MOESM1_ESM.pdf]
